# Impact of Citral and Phloretin, Alone and in Combination, on Major Virulence Traits of *Streptococcus pyogenes*

**DOI:** 10.3390/molecules24234237

**Published:** 2019-11-21

**Authors:** Mohd Adil, Mohd Hassan Baig, H.P. Vasantha Rupasinghe

**Affiliations:** 1 Department of Plant, Food, and Environmental Sciences, Faculty of Agriculture, Dalhousie University, Truro, NS B2N 5E3, Canada; mohd.adil@dal.ca; 2 Department of Family Medicine, Gangnam Severance Hospital, Yonsei University College of Medicine, 211 Eonju-ro, Gangnam-gu, Seoul 06273, Korea; mhbaig@ynu.ac.kr; 3 Department of Pathology, Faculty of Medicine, Dalhousie University, Halifax, NS B3H 4H7, Canada

**Keywords:** Group A streptococcus, phytochemicals, antibiotic resistance, biofilm, quorum sensing, LuxS protein

## Abstract

*Streptococcus pyogenes* is well documented as a multi-virulent and exclusively human pathogen. The LuxS-based signaling in these bacteria has a crucial role in causing several infections through pathways that are pathogenic. This study evaluated the individual and synergistic effects of citral and phloretin against *S. pyogenes* in relation to major virulence traits. The in vitro synergy of citral and phloretin was evaluated by the checkerboard method. The fractional inhibitory concentration (FIC) values were calculated to determine the interactions between the inhibitors. The bacteria’s virulence properties were tested in the presence of the molecules, individually as well as in combination. Molecules’ cytotoxicity was tested using human tonsil epithelial cells. The synergistic effects of the molecules on the expression of biofilm and quorum sensing genes were tested using quantitative real-time polymerase chain reaction (qRT-PCR). The molecules were also tested for their impact on LuxS protein by molecular docking, modeling, and free-energy calculations. When the two molecules were assessed in combination (synergistic effect, FIC Index of 0.5), a stronger growth inhibitory activity was exhibited than the individual molecules. The cell surface hydrophobicity, as well as genes involved in quorum sensing and biofilm formation, showed greater suppression when the molecules were tested in combination. The *in silico* findings also suggest the inhibitory potential of the two molecules against LuxS protein. The binding orientation and the binding affinity of citral and phloretin well support the notion that there is a synergistic effect of citral and phloretin. The data reveal the combination of citral and phloretin as a potent antibacterial agent to combat the virulence of *S. pyogenes.*

## 1. Introduction

One of the leading causative agents of acute bacterial pharyngitis, also called strep throat, is *Streptococcus pyogenes* (Group A, GAS). The prevalence of this infection is reported to be 15–36% in children and 5–15% in adults [1]. Annual global new cases of pharyngitis are more than 616 million. Therefore, *S. pyogenes* infection is one of the major contributors to health care expenses [2]. Conditions such as post-streptococcal glomerulonephritis (kidney inflammation) and rheumatic fever due to acute GAS infections, in children, may lead to disability and death [3]. In under-developed countries, the primary cause of cardiovascular deaths was reported to be associated with rheumatic heart disease or rheumatic fever [4].

The progression of GAS infectious diseases involves several essential steps, starting with competition between the bacteria and the normal flora of nasopharynx to adhere to host tissues. Cell aggregates are formed by the interaction between the extracellular matrix, host cells, salivary glycoproteins, and other microbes, which leads to proliferation and formation of microcolonies. The last stage involves disease progression, where microcolonies form distinct communities that are encapsulated by the exopolysaccharides (EPS) and termed as mature biofilms [5,6]. Patients suffering from a number of medical conditions, including atopic dermatitis, strep throat, glomerulonephritis, impetigo, etc., have shown the presence of microcolonies [3]. The pathogenesis of GAS has been reported to be majorly influenced by the development of biofilm in the soft tissues of the host, such as skin, throat, and heart valves [4]. The role of biofilms contributing to the pathogenesis of GAS has been extensively studied [5]. M protein plays a paramount role in the establishment of cell-surface interaction in biofilms, while the hyaluronic capsule leads to biofilm maturation. The bacteria through quorum sensing (QS), releases specific chemical signals that regulate the expression of virulence genes activities within biofilms [7].

The preferred antibiotics for the treatment of *S. pyogenes* infections are β-lactams, such as penicillin [8]. However, recent cases of antibiotic treatment failures against *S. pyogenes* have emerged. One of the reasons for the antibiotic resistance is the over-prescription of antibiotics to treat pharyngitis [9]. Another mechanism in developing multi-drug resistance (MDR) is through the efflux pumps that pump out the antibiotics by enhancing resistance in bacteria [10]. Thus, it is necessary to find new agents with the ability to treat infectious diseases while being less toxic to humans. Pharmaceutical companies have all but removed themselves from antimicrobial drug discovery; therefore, alternative therapeutic approaches are needed [11]. There have been continuous efforts to find new drugs that may possess antimicrobial potential from natural sources such as phytochemicals. Plant parts such as bark, fruits, leaves, seeds, and roots have been used to extract unique phytochemicals that may contain antimicrobial compounds [12].

Citrus oils mainly contain 3,7-dimethyl-2-6-octadienal, which is called citral. It is reported to have anti-inflammatory [13] and bactericidal properties [14]. It has also been reported to exert antifungal activity against yeasts [15]. Citral acts by disrupting the fungal cell membrane, thereby compromising its permeability and integrity [16]. Citral has been reported to function as an efflux pump inhibitor in *S. aureus* [17]. Phloretin, a polyphenolic chalcone, has many interesting biological properties, including inhibition of Gram-positive and Gram-negative bacteria. Fruit flavonoids containing phloretin have been reported to modulate efflux pumps in *S. aureus* by reducing the norfloxacin resistance in this bacterium [18].

Quorum sensing (QS) is a mechanism by which many bacterial species modulate the gene expression based on their cell population density [19,20,21]. Bacterial survival approaches include host cell colonization, ability to withstand different physical conditions, and developing defense mechanisms against other microbes as well as a range of anti-microbial agents that act via different mechanisms of action [22]. Gram-positive bacteria secrete peptides called autoinducers that act as binding sites or ligands for the cell’s signal receptors. Autoinducer-2 (AI-2) is a signaling molecule that is synthesized by LuxS protein, which is involved in the QS process. LuxS plays an important role in the virulence properties of *S. pyogenes*, such as the ability to form biofilm [23]. A study conducted by Lyon and colleagues involved mutation of *luxS* gene, which resulted in disrupted virulence activity, increased expression of *sagA* gene, and reduced proteolytic activity of SpeB. In another study on mutated *luxS* gene, Kang et al. reported a noteworthy reduction in the secretion of the virulence gene SpeB and an altered in vitro growth rate in the bacteria [24]. These studies revealed the indispensable role of LuxS in *S. pyogenes* virulence [25].

The effects of citral and phloretin have been demonstrated against various microbes. However, their effects on the activity of *S. pyogenes* and possible modes of action have not been studied. Our study is the first attempt to report the impact of these molecules alone as well as synergistically (i.e., in combination) on the virulence properties and gene expression of *S. pyogenes*. The important role of LuxS in bacterial systems, as well as its absence in the human genome, makes it one of the potential therapeutic drug targets [19,24,25,26,27]. Therefore, our study also explored the interaction of citral, phloretin, and their combination with LuxS protein using *in silico* approaches.

## 2. Results

### 2.1. Determination of the Minimum Inhibitory Concentration (MIC) and the Fractional Inhibitory Concentration (FIC) Index Values

**Figure 1:**
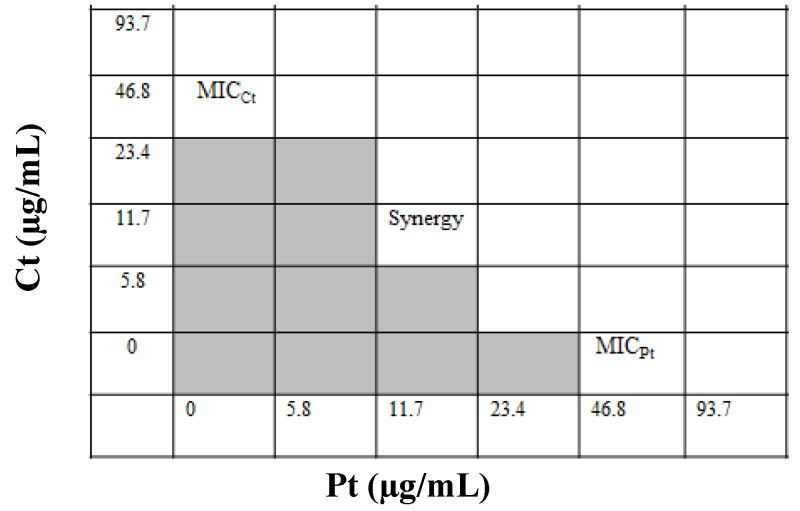
Assessment of the synergistic antibacterial effect of citral (Ct) and phloretin (Pt) by the checkerboard method (shading: visible growth).

The individual MICs of Ct and Pt against *S. pyogenes* were 46.8 µg/mL. To determine FIC index value, a checkerboard assay was performed in which various concentration combinations of Ct and Pt (ranging from several dilutions below the MIC to twice the MIC) were assessed against *S. pyogenes*. As evident in Figure 1, the best FIC index for the combination (Ct + Pt) was the one with the lowest value, which was found to be 0.5.

### 2.2. Cytotoxicity Assay

**Figure 2:**
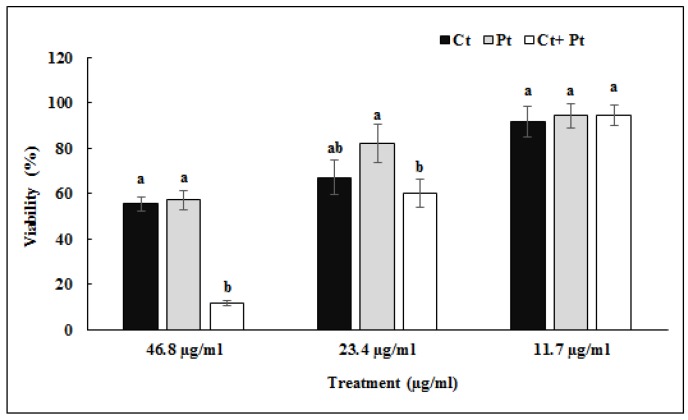
Effect of citral (Ct), phloretin (Pt), and their combination (Ct + Pt) on the viability of tonsil epithelial cells as measured by MTS assay (values are mean ± SD, n = 3). Bars (means) with different letters (a and b) are significantly different from others (*p* < 0.05).

The cytotoxicity of both Ct and Pt on human tonsil epithelial cells (HTonEpiC) was evaluated by the 3-(4,5-dimethylthiazol-2-yl)-5-(3-carboxymethoxyphenyl)-2-(4-sulfophenyl)-2H-tetrazolium (MTS) assay. The tonsil epithelial cells were incubated 24 h with both molecules, individually as well as in combination, at varying concentrations ranging 0–46.8 µg/mL. Ct showed cytotoxic effects at a concentration higher than 23.4 µg/mL, while a similar effect by Pt was shown at the concentration of 46.8 µg/mL. No cytotoxic effect was observed on tonsil epithelial cells when the molecules were used in combination with the concentration of 11.7 µg/mL of both molecules (Figure 2).

### 2.3. Effect of Ct and Pt Alone and in Combination on the Growth of S. pyogenes

**Figure 3:**
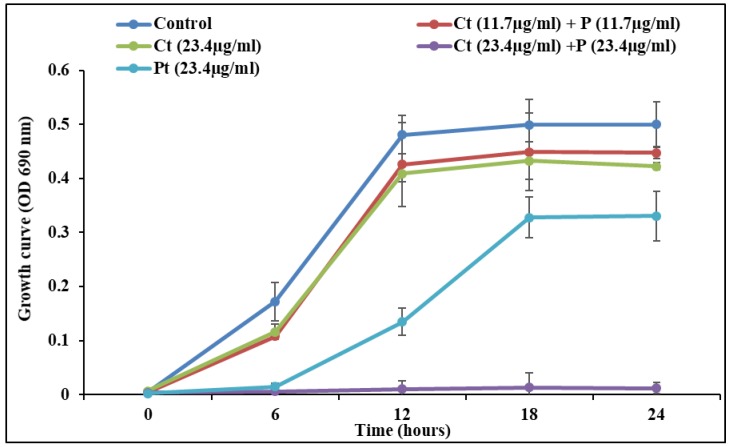
*S. pyogenes* growth curve at a sub-MIC concentration of citral (Ct), phloretin (Pt), and their combination (Ct + Pt). The experiment was performed thrice, and the wavelength of the spectrophotometer was set at 690 nm.

*S. pyogenes* growth pattern was measured in the presence of test compounds alone and combination. It was found that all treatments showed a typical sigmoidal growth curve. However, the variation in the growth pattern between control and combination (Ct + Pt) was least. The data obtained validate that this concentration of synergy Ct + Pt (11.7 µg/mL + 11.7 µg/mL) does not exhibit a bactericidal effect (Figure 3).

### 2.4. Effect of Ct and Pt (alone and in combination) on S. pyogenes Biofilm Formation and Cell Surface Hydrophobicity

**Figure 4:**
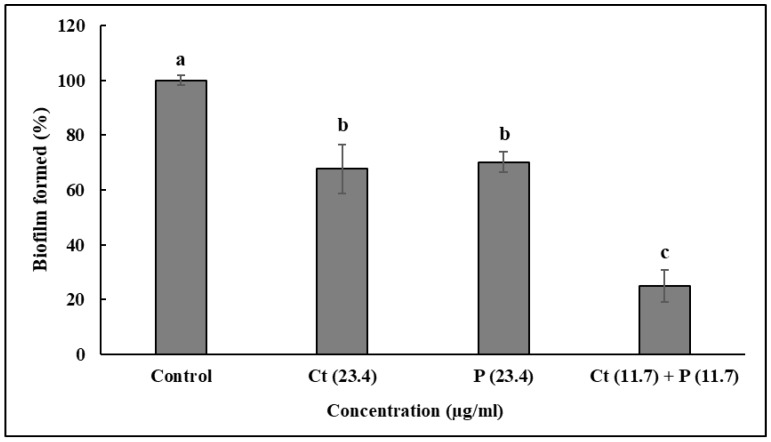
Effect of citral (Ct: 23.4 µg/mL) and phloretin (Pt: 23.4 µg/mL) and their combination (Ct + Pt: 11.7 µg/mL + 11.7 µg/mL) on biofilm formation when the bacteria was grown for 24 h in the presence of molecules. Bacteria grown under similar conditions without any test molecules were taken as the control. Data represent mean ± SD from three independent experiments. Bars (means) with different letters (a–c) are significantly different from others (*p* < 0.05).

**Figure 5:**
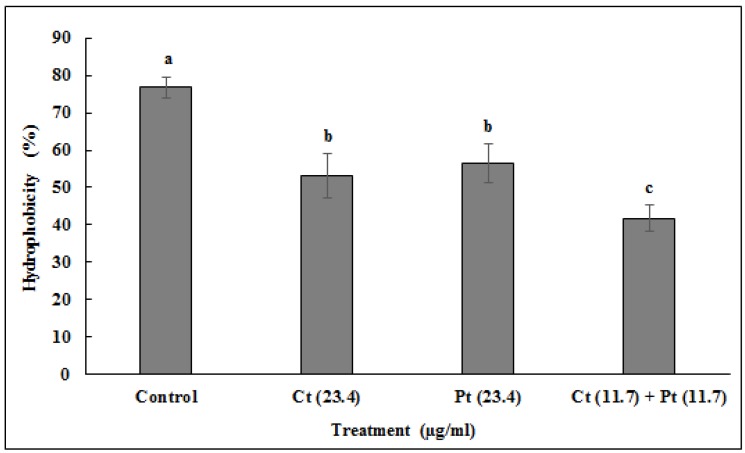
Inhibitory effect of citral (Ct: 23.4 µg/mL) and phloretin (Pt: 23.4 µg/mL) and their combination (Ct + Pt: 11.7 µg/mL + 11.7 µg/mL) on *S. pyogenes* cell surface hydrophobicity. The experiments were carried out in triplicate (values are mean ± SD, n = 3). Bars (means) with different letters (a–c) are significantly different from others (*p* < 0.05).

The anti-biofilm effect of Ct, Pt at 23.4 µg/mL each and Ct + Pt at 11.7 µg/mL is presented in Figure 4. The combination of Ct + Pt displayed the most appreciable decrease in the ability of *S. pyogenes* to form biofilm. After 24 h, when compared to the control, the maximum decrease in biofilm development in the presence of Ct, Pt, and Ct + Pt was 48.66%, 46.39%, and 67.57%, respectively. The adhesion of *S. pyogenes* cells to toluene in the presence and absence of the molecules was used to measure the hydrophobicity of test molecules (Figure 5). Ct + Pt combination caused a significant reduction in hydrophobic interactions compared to when molecules were tested individually. Adhesion to toluene decreased from 76% in the untreated control to 53.6%, 55%, and 41.2% on treatment with Ct, Pt, and Ct + Pt, respectively.

### 2.5. Confocal Laser Scanning Microscopy (CLSM) Analysis

**Figure 6:**
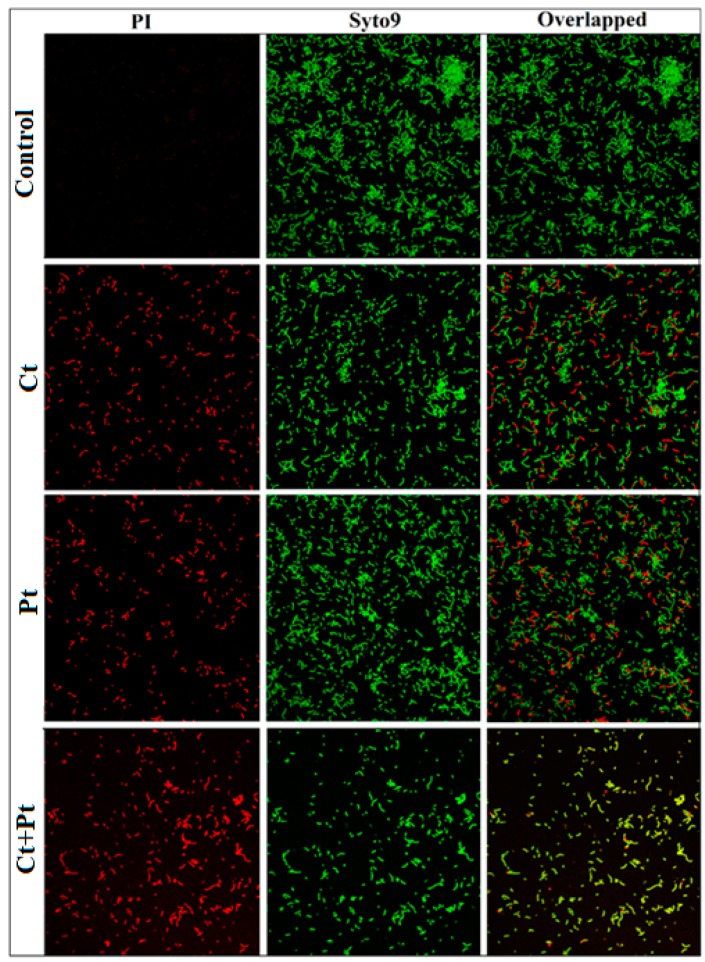
Confocal Laser Scanning Microscopy images of *S. pyogenes* biofilms in the absence of any compound (Control) and upon treated with citral (Ct, 46.8 µg/mL) and phloretin (Pt, 46.8 µg/mL) and their combination (Ct + Pt: 23.4 µg/mL + 23.4 µg/mL). Live cells and dead cells were stained with SYTO 9 (green) and propidium iodide (red/yellow). Scale bars = 20 µm. The images are representative of three independent experiments.

The results of MIC and checkerboard assay were found to agree with the observations made in the CLSM images. *S. pyogenes* cells were grown in the presence of both the molecules individually as well as in combination and were visualized by CLSM using SYTO 9 (green) and PI (red/yellow) that stained live cells and dead cells, respectively. The combination (Ct + Pt) exerted a higher deteriorating impact than Ct and Pt alone on cell aggregation and cell-viability of *S. pyogenes*. As shown in Figure 6, the maximum dispersion of cells within the matrix was evident in the case of the treatment with the combination of Ct and Pt.

### 2.6. Impact of Ct, Pt, and Ct + Pt on Gene Expression

**Figure 7:**
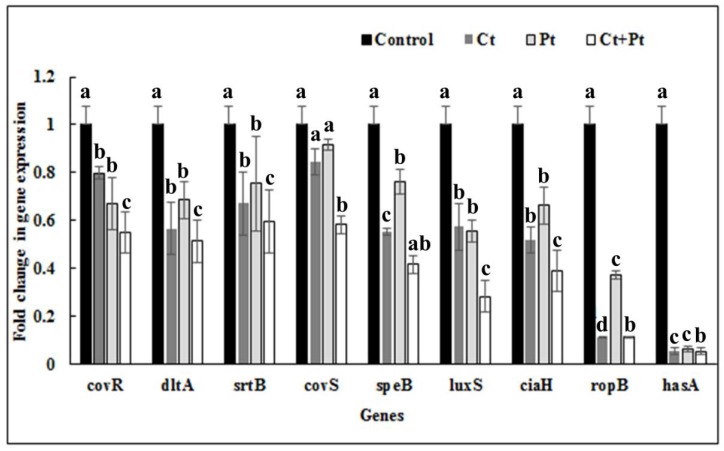
The *S. pyogenes* virulence genes expression profile in the presence of citral (23.4 µg/mL Ct), phloretin (23.4 µg/mL Pt), and their combination (11.7 µg/mL Ct + 11.7 µg/mL Pt). Bacteria grown under similar conditions without any test compound were taken as control. The data were obtained from triplicate sets of experiments (data are presented as mean ± SD). Bars (means) with different letters (a–c) are significantly different from others (*p* < 0.05).

The impact of Ct, Pt, and their combination on the expression of virulence genes of *S. pyogenes* was tested using qRT-PCR. The genes included those involved in biofilm formation, QS, and other important virulence attributes of this microbe. To reveal the transcript level of these genes in the absence and presence of the test molecules, the total RNA from early exponential-phase cultures were used. The combination Ct + Pt exhibited a maximum reduction in the expression of the *ropB* gene and *hasA* genes, i.e., >80%, while the expression of *speB, dltA, luxS,* and *ciaH* genes was suppressed >60% when compared to the control. It was also revealed that Ct was capable of negatively affecting the expression of *covR, dltA, srtB, speB, luxS, ciaH*, and *ropB* genes by 20.3%, 33.6%, 32.9%, 44.7%, 42.7%, 48.2%, and 66.9%, respectively. Similarly, Pt effectively suppressed the expression level of *covR, dltA, srtB, speB, luxS, ciaH,* and *ropB* genes by 33.1%, 31.6%, 24.7%, 23.8%, 44.5%, 33.8%, and 62.8%, respectively. Evidently, both Ct and Pt suppressed the *luxS* gene by 42.7% and 44.5%, respectively (Figure 7).

### 2.7. Molecular Docking Studies

**Figure 8:**
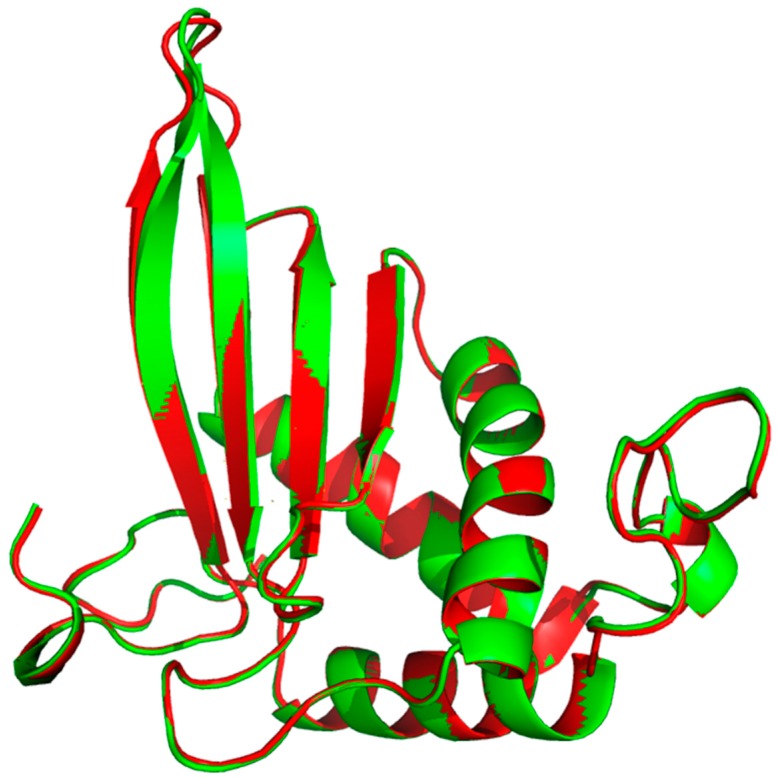
Superimposition of the modeled structure of LuxS from *S. pyogenes* (Red) and LuxS from *S. suis* (Green).

**Figure 9:**
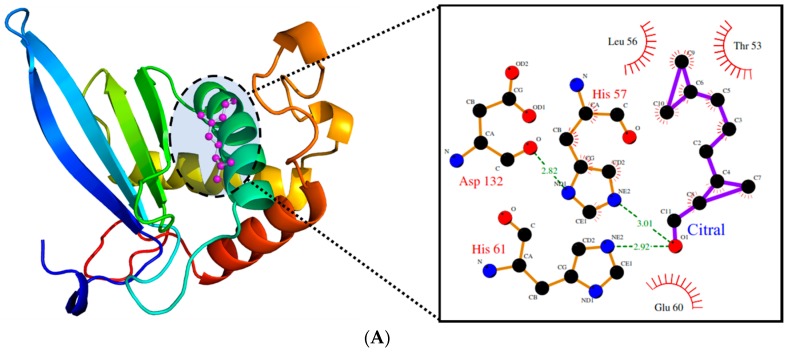

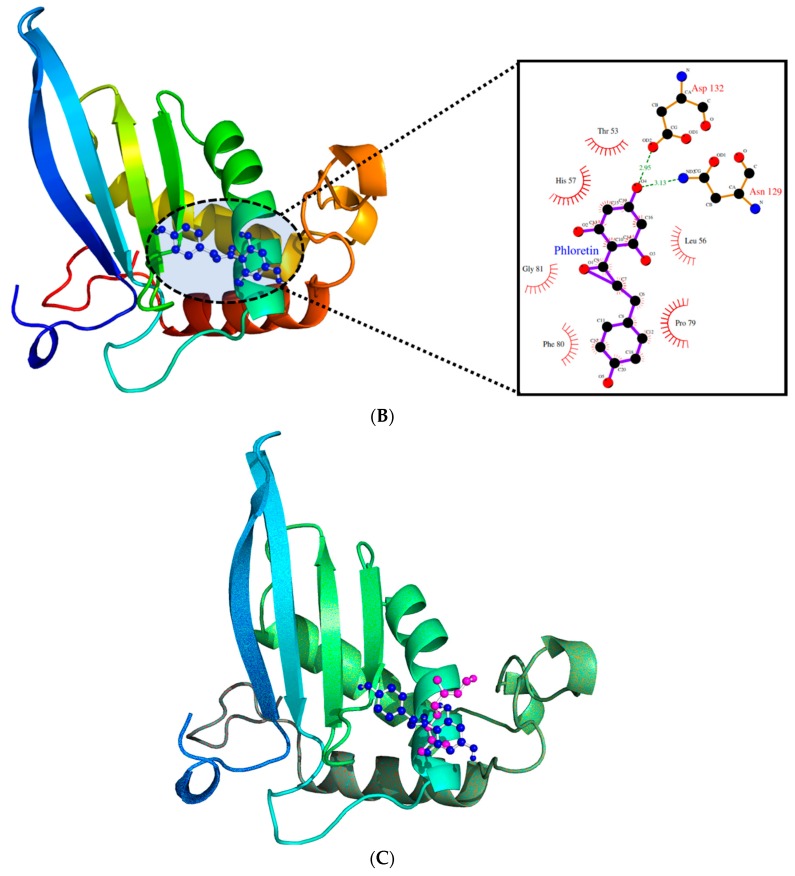
(**A**). The binding pattern of citral within the active site of LuxS. (**B**). The binding pattern of phloretin within the active site of LuxS. (**C**). The cluster showing the binding pattern of both citral (magenta) and phloretin (blue) within the active site of LuxS.

Due to its major role in the pathogenicity of *S. pyogenes,* docking of the test molecules (alone and in combination) into the binding pocket of LuxS from *S. pyogenes* was carried out. The homology model of *S. pyogenes* protein LuxS was generated using the crystal structure of LuxS from *Streptococcus suis* (pdb id: 4XCH) as the template, showing 81% similarity. The superimposition of the modeled structure of *S. pyogenes* protein, LuxS, and the template structure showed the root-mean-square distance (RMSD) of 0.083 Å (Figure 8). Furthermore, validation of the quality of the modeled structure of LuxS and the confirmation of its stereochemical properties was performed using the software Procheck. It was revealed through the Ramachandran plot that 97.9% of the total residues of the modeled structure of LuxS were in the most favored regions while 3% were in the allowed region, and there were no residues in the outlier or disallowed region. Molecular docking study of Ct and Pt was performed against the modeled structure of LuxS. For the protein–ligand complex, the binding free energy of these molecules against LuxS was calculated. The binding free energies that existed during the interaction with Ct and Pt were of the value −3.82 and −4.97 kcal/mol, respectively (Table 1). The binding of both molecules within the active site of LuxS was dominated by hydrophobic interactions. T53, L56, and H57 were investigated to be common residues involved in the positioning of Ct and Pt within the binding pocket of LuxS (Figure 9).

## 3. Discussion

Natural antibacterial agents such as specific phytochemicals and their combinations have recently gained popularity as an alternative approach to combat antibiotic-resistant bacteria. No report has been published related to the potential of Ct, Pt, or both combined against *S. pyogenes.* The current study suggests the potent role of Ct and Pt in combination, synergistically inhibiting a range of virulence-related properties of *S. pyogenes* as compared to their effect individually. Ct and Pt both exhibit a substantial antimicrobial activity with a low-moderate MIC value of 46.8 µg/mL each. It was previously shown that the MICs of plant-derived molecules (myricetin, phlorizin, and cinnamaldehyde) against *S. pyogenes* ranged from 50 to 100 µg/mL [6]. FIC index of ≤ 0.5 represents a good predictor of synergy, which was found to be 0.5 for Ct and Pt. This is in line with the synergy study conducted on *S. pyogenes* using plant-based compound carvacrol and erythromycin [28]. Cytotoxicity of Ct, Pt, and Ct + Pt on tonsil epithelial cells was evaluated by MTS assay. If tonsil epithelial cells are destroyed or negatively impacted by treated molecules, then they are rendered useless in developing novel anti-*S. pyogenes* agents that could be incorporated in orally taken natural health products for the management of infections associated with this bacterium. Ct + Pt at a concentration of 11.7 µg/mL showed significantly higher cell viability (>80%), which indicates the safe use of the combination [29]. Upon the observation of the growth curve of treated samples and control, it was revealed that Ct and Pt are not toxic to *S. pyogenes* when tested at a concentration of 11.7 µg/mL for both compounds (Figure 2). The result shows that Ct + Pt at tested concentration does not exhibit any bactericidal activity against *S. pyogenes*, which adds to their value as an ideal anti-biofilm agent. Therefore, compared to antibiotics, they will be less prone to resistance development.

One of the approaches to develop efficient anti-*Streptococcal* agents is to target their ability to spread infection through adherence. The interplay between hydrophobic interactions of the bacterial cells with those of the host cells results in strong adherence, thus distorting this hydrophobic interaction would cause a reduction in adherence [30,31]. This may happen either due to the loss of the adhesin protein from the cell surface or as a result of any modification that may lead to suppression of the bacterial adhesin (ligand on the bacterial surface) that mediates adherence [32]. There was an apparent reduction observed in the cell surface hydrophobicity of *S. pyogenes* in the presence of tested molecules. It has been shown that Ct reduced hydrophobicity by 29.4% while Pt reduced it by 27.6%. However, Ct + Pt showed a collective activity by decreasing hydrophobicity significantly to a lower level (45.8%) than they decreased individually, thus indicating their synergistic effect.

CLSM revealed the impact of the compounds on the ability of *S. pyogenes* to thrive (Figure 6). The mixture of the two fluorescent nucleic acid stains used, i.e., PI (red/yellow, dead cells) and SYTO 9 (green, live cells), revealed that Ct + Pt in combination could cause membrane damage in *S. pyogenes* [33], as demonstrated by the existence of red/yellow fluorescent bacterial cells within the Ct + Pt treated biofilm. The CLSM images revealed that the membrane of *S. pyogenes* was compromised in the treated sample, whereas the control sample showed the aggregated cells, as was evident by the presence of many green-fluorescent bacteria. Based on these results, Ct + Pt appears to be an ideal combination to prevent *S. pyogenes* infection. Adil et al. [34] conducted similar microscopy analysis using plant-based molecule eugenol and observed a similar pattern in *S. mutans*.

Table 2 lists some of the important genes reported playing an indispensable role in *S. pyogenes* biofilm formation and its virulence. The qPCR quantification of these genes was carried out to elucidate the possible mechanism for the reduction in biofilm formation and also to study the effect of Ct, Pt, and Ct + Pt on virulence factors. It was found that all of these genes were down-regulated in the presence of Ct and Pt individually. However, maximum repression was observed in the synergistic combination (Ct + Pt). The tested compounds inhibited aggregation of *S. pyogenes*, which is believed to be due to the combined effect of down-regulation of *srtB* (transpeptidase) and *hasA* (hyaluronic acid production) genes.

The genes responsible for the expression of RopB and HasA protein were profoundly repressed (Figure 6). *ropB* expresses a transcriptional regulating protein that influences other genes that play a critical role in virulence, stress response, and metabolism in these bacteria [35]. The gene *hasA* synthesizes hyaluronic acid (HA) capsule that helps *S. pyogenes* to evade the immune response of the host [36]. Betulin, a plant-based compound, showed a similar impact on *ropB* gene. However, the repression of *hasA* was a mere 9% reduction, which contradicted the results of this study [37]. The genes of the *covRS* TCS pathway, namely *covR* (repressor) and *covS* (sensor kinase), were moderately down-regulated in the presence of Ct + Pt. *covRS* TCS pathway influences nearly 15% of genes, including those playing a pivotal role in quorum sensing and hyaluronic acid synthesis in *S. pyogenes* [23,36]. Ct and Pt possess antioxidant properties [38,39]. The oxidative stress response in *S. pyogenes* requires the expression of the gene *CiaH.* Therefore, the influence of Ct and Pt was assessed individually as well as in combination. Ct + Pt treatment was found to down-regulate *ciaH* expression significantly. This gene is believed to participate in the early stages of infection, but it is not important in the later stages of infection that leads to its virulence [40].

Neutrophils migrate to *S. pyogenes* in severe invasive infections and are reported to release neutrophil extracellular traps (NET). NET is fragmented by *speB* (streptococcal erythrogenic toxin B), expressed by this bacterium, which contributes to the destruction of host tissues and matrix proteins [41]. It further helps the bacteria to escape the attack of neutrophils by inactivating C3b (a protein of innate immune system) at the initial infection stage [42]. In our study, this gene was found to be down-regulated by 58.6%. As shown in Figure 7, there was 58.8% down-regulation of *dltA* gene observed in the samples treated with Ct + Pt. One of the important cell wall components in Gram-positive bacteria is D-alanine incorporated lipoteichoic acid (LTA). The gene *dltA* is responsible for D-alanylation of LTA that helps in enhancing cell surface hydrophobicity in *S. pyogenes* [43,44]. This is in line with the results of the hydrophobicity assay conducted in the presence of test compounds. Thus, down-regulation of *dltA* can be attributed as one of the reasons for reduced biofilm formed in Ct + Pt treated *S. pyogenes*. Nandu et al. [45] demonstrated similar reduction in the expression of *dltA*, *covS* and *hasA* genes using the plant-based compound fukugiside. However, as opposed to our findings, the *speB* gene was up-regulated upon the treatment with fukugiside. In another study, botulin, a plant terpenoid, caused down-regulation of *ropB*, *srtB* and *dltA* [37]. Similar to the observation in our study, it was reported that inactivation of the *luxS* gene causes a reduction in *speB,* which is responsible for extracellular protease [37]. This observation is supported by LuxS mutation in *S. pyogenes* significantly reducing the mRNA level of speB [46].

The recent studies aiming at finding the treatment of bacterial infections have attracted the attention of researchers toward targeting the LuxS-based QS system [46]. Reports suggest the omnipresence of LuxS in bacterial systems could make it an important therapeutic drug target, and, therefore, many inhibitors against LuxS have been investigated recently [47]. These screening processes were performed using traditional and modern computer-aided virtual screening methods [47,48,49]. In the present study, LuxS was selected as the target protein because of its ability to synthesize the autoinducer-2 (AI-2) signaling molecules, which are responsible for inter/intraspecies quorum sensing (QS) response. The AI-2 signaling molecules are important components of the machinery involved in controlling various cellular processes.

The binding confirmation and the affinity between ligand and its target protein can be initially and efficiently investigated by molecular docking studies. In our study, molecular docking of Ct and Pt with LuxS (target protein) was carried out, and it was found that these test molecules were carrying inhibitory potential against LuxS, by displaying good inhibitory activity (in terms of binding energy). The binding orientation of both molecules was found to differ in the binding pattern (Figure 9). Both Ct and Pt formed stable conformations within the LuxS active site and were interacting with the binding free energy of −3.82 and −4.97 kcal/mol, respectively (Table 1). The binding affinity of both molecules was almost the same, and both were found to anchor in the same conformation (Figure 9C), thus implying that both molecules are accountable for the activity. Therefore, based on *in silico* results, it can be suggested that the inhibitory activity of LuxS is because of the synergistic effect of both the molecules.

Overall, in vitro and in silico results of this study suggest a potential use of Ct and Pt against *S. pyogenes*. In addition, there is evidence of synergism between these two compounds, suggesting the possible use of these molecules in combination for exerting desirable results at concentrations as low as 11.7 µg/mL, which is a two-fold lower concentration than that of the molecules individually. Thus, these molecules might be useful against *S. pyogenes*, which cause several infectious diseases in humans. In the future, exploring the efficacy and safety of Ct and Pt using human subjects would help in developing novel therapeutics against infectious diseases caused by *S. pyogenes*.

## 4. Materials and Methods

### 4.1. Test Bacterium, Cell Line, and Molecules

*S. pyogenes* ATCC 19615 was purchased from American Type Culture Collection (ATCC, Manassas, VA, USA). It was isolated from the pharynx of a child following episode of strep throat. The bacteria were grown for 24 h at 37 °C in Brain Heart Infusion (BHI) broth (Oxoid, Thermo Fisher Scientific, Waltham, MA, USA). The tonsil epithelial cells (ScienCell Research Laboratory, San Diego, CA, USA) were grown and maintained in poly-l-lysine (PLL) coated 2 mg/cm2 T-75 flask at 37 °C and 5% CO_2_ with complete growth medium (prepared using growth supplement, penicillin/streptomycin solution and tonsil epithelial cell medium at 1:1:100 ratios), as described by Wijesundara et al. [29]. Citrals, phloretin, and all tested chemicals were obtained from Sigma-Aldrich (Saint-Louis, MO, USA). The purity of compounds was ≥96%. The stock solutions (3 mg/mL) were freshly prepared in DMSO for the experiments. (Catalogue no. Ct: C83007, Pt: P7912)

### 4.2. Bacteriostatic and Bactericidal Concentrations

The double dilution method, as described previously [50], was used to establish the MIC (bacteriostatic) of the molecules against *S. pyogenes.* The bacteria were treated with different concentrations of the compounds (0.36 to 750 µg/mL) in a series of double dilutions. The MIC was the lowest concentration that completely inhibited visible bacterial growth. The plates were then incubated for 24 h at 37 °C. DMSO (0.05%) and nimesulide (0.5–5 µg/mL) were used as negative and positive control, respectively. The tests were repeated thrice, and all determinations represented the mean of three independent experiments.

### 4.3. Checkerboard Test

Synergy was examined with the help of the checkerboard method. The effect of citral (Ct) and phloretin (Pt) combination was assessed by serial two-fold dilutions of each molecule using a 96-wells microtiter plate. Fifty microliters of BHI broth were dispensed in each well of the microtiter plate. First, citral was serially diluted along the ordinate, followed by the addition of phloretin, which was diluted along the abscissa. To each well of a microtiter plate, 50 µL of bacterial inoculum of 10^5^–10^6^ CFU/mL was added, and the plates were incubated for 24 h at 37 °C under anaerobic conditions. This was followed by the determination of indifferent or antagonistic FIC index, to evaluate the effect of the combination to be synergistic [28]. The FIC index of the combination was calculated by the formula:

FIC index = FIC_Ct_ + FIC_Pt_ (1)

where FIC_Ct_ is the MIC of Ct in combination/MIC of Ct alone and FIC_Pt_ is the MIC of Pt in combination/MIC of Pt alone. FIC index value of ≤0.5 is considered to represent a synergy between the combinations while indifference or absence of interaction is indicated by a value of >0.5 to <4, and antagonistic when the FIC index is ≥4.

### 4.4. Cell Viability Assay

The MTS assay described by Wijesundara et al. was carried out to determine cell viability of tonsil epithelial cells in the presence of tested compounds [29]. Each of the 96 wells of the microtiter plate was pre-coated with poly l-lysine (PLL) at a density of 5000 cells/100 µL and treated with Ct, Pt, and Ct + Pt at concentrations of 5.8–46.8 µg/mL. DMSO (0.05%) and nimesulide (0.5–5 µg/mL) were used as negative and positive control, respectively. Plates were incubated at 37 °C for 24 h. After incubation, 20 µL of MTS reagent (MTS + phenazinemethosulfate) were added and again incubated for another 2.5 h. The absorbance was quantified by reading at 490 nm. The cell viability was calculated as:

% cell viability = (A_490treated wells_ − A_490blank_/A_490control wells_ − A_490blank_) × 100 (2)

### 4.5. Growth Curve Assay

Growth curve assay was performed in microtiter plates containing BHI, bacterial inoculum, and different combinations of test compounds [i.e., sub-MIC of Ct (23.4 µg/mL), Pt (23.4 µg/mL), and their combinations Ct + Pt (23.4 µg/mL + 23.4 µg/mL), and Ct + Pt (11.7 µg/mL + 11.7 µg/mL)]. One of the controls included an equivalent reaction mixture containing 0.1% DMSO. The plates were incubated at 37 °C, statically. The culture’s spectrophotometry readings (OD_690_) were recorded, and the survival rates were obtained at predetermined time intervals of 0, 6, 12, 18, and 24 h after incubation [28]. All experiments were carried out in triplicate.

### 4.6. Biofilm Formation Assay

Biofilm formation assay was done by the protocol recently described by Zutkis et al. [51]. It was performed in cell-culture-treated 24-well polystyrene plates containing BHI, bacterial inoculum, and with different combinations of test compounds, as described above. The diluted culture (1:100) of *S. pyogenes* grown overnight was added to fresh BHI broth and incubated for approximately 1 h at 37 °C until they reached an OD_600_ ~ 0.02 and then inserted in triplicates. This was followed by the plate’s incubation at 37 °C for 24 h in 5% CO_2_ to promote biofilm growth. After incubation, the culture was aspirated from the wells and washed thrice with 200 µL of phosphate-buffered saline (PBS) to remove the planktonic cells. Then, the plate was heat fixed for 1 h at 60 °C. The adhered bacterial cells were stained with 200 µL of crystal violet (0.1%) for 2 min. After staining, the wells were water rinsed at least thrice. Then, 200 µL of destain solution, prepared by mixing 7.5% acetic acid and 10% methanol in distilled water, was added. DMSO (0.05%) and nimesulide (0.5–5 µg/mL) were used as negative and positive control, respectively. The absorbance was measured at 540 nm after the plates were shaken for 1 min.

### 4.7. Microbial Adhesion to Hydrocarbon (MATH) Assay

The estimation of the impact of molecules on the cell surface hydrophobicity of *S. pyogenes* was done using MATH assay. It was also used to determine the adhering capability of the bacterial cell to hydrophobic substrates [52]. Toluene (100 µL) was added to the glass tubes containing 1 mL of bacteria (OD_530_ = 1.0). The phase separation was established by vigorously vortexing the tubes for 2 min, and incubating the tubes at room temperature for 10 min. The absorbance of the lower aqueous phase was noted spectrophotometrically at 530 nm. The hydrophobicity of the bacterial cell surface was calculated by the equation:

Percent (%) Hydrophobicity = [1 − (OD_530_ after vortexing/OD_530_ before vortexing)] × 100 (3)

### 4.8. CLSM Analysis of S. pyogenes

CLSM was performed to observe the effect of tested compounds on one-day-old cell culture. *S. pyogenes* was cultured in BHI broth at 37 °C for 24 h consisting of a coverslip in each well of 12-well microtiter plate (Nunc, Rochester, NY, USA). One-day-old cells attached to the coverslip were treated with test molecules of varying concentrations and incubated for 24 h at 37 °C. Normal saline was used to remove media from the coverslips and then stained with SYTO 9 and propidium iodide mixture (LIVE/DEAD Biofilm Viability Kit, Molecular Probes, Invitrogen, Nepean, ON, Canada) for 20 min in the dark. The coverslips were placed under a laser scanning confocal imaging system with an excitation wavelength of 480/500 nm (Zeiss LSM 510 META; Heidelberg, Germany) attached with a water immersion dipping objective lens of 60× oil. This was followed by image capturing of the cells with an inverted microscope attached to the CLSM imaging system (Zeiss LSM 510 META; Heidelberg, Germany). Three positions were randomly selected, and this was followed by an examination of each stack. The threshold value that presented the best fit for all the image stacks of a trial was chosen [53].

### 4.9. RNA Extraction and Synthesis of cDNA

*S. pyogenes* cells treated with Ct (23.4 µg/mL), Pt (23.4 µg/mL), and Ct + Pt (11.7 + 11.7) µg/mL, or untreated (control), were grown at 37 °C in BHI medium for 24 h. RNeasy Mini kit (Qiagen, Toronto, ON, Canada) was used to process the cell for RNA isolation. The total RNA concentration was determined using a Nano-Quant Plate, TECAN (Infinite M200 PRO, TECAN, Atlanta, GA, USA). The integrity of the isolated RNA was confirmed by running agarose gel electrophoresis. This was followed by the removal of contaminating genomic DNA by the addition of DNase I (Bio-Rad) prior to cDNA synthesis. One microgram of total RNA was taken to synthesize cDNA using an i-Script g DNA clear cDNA Synthesis Kit (Bio-Rad, Mississauga, ON, Canada) as per the manufacturer instructions. The conditions included the following: a cycle of 5 min at 25 °C; 20 min at 46 °C; and 1 min at 95 °C. The freshly prepared cDNA was immediately used for quantitative real-time PCR (qRT-PCR) [54].

### 4.10. Expression of Quorum Sensing and Biofilm Formation Genes

The Fasta format sequence of genes was downloaded from the National Center for Biotechnology Information (NCBI) website. The primer sequences of genes mentioned in Table 2 were designed using the algorithms provided by Primer quest tool (Integrated DNA Technologies, Coralville, IA, USA) for uniformity in size (ca. 95 bp) and melting temperature. A final volume of 20 µL was taken to carry out qRT-PCR in a 96-well PCR plate (Bio-Rad, Cat. No. HSP9601) using CFX Connect System (Bio-Rad) and Sso-Advanced Universal SYBR Green PCR super-mix (Bio-Rad). The cycling conditions were set at defaults which were 2 min at 94 °C; 10 min at 95 °C; and 40 cycles of 15 s at 95 °C, and 1 min at 60 °C. The experiment was repeated thrice, which included three technical replicates each time. All experiments included the transcript encoding gyrA served as the gene for calibration. The 2^−ΔΔCt^ method was used to calculate expression ratios [55].

### 4.11. In silico Studies

#### 4.11.1. Molecular Modeling

The 3D structure of LuxS was unavailable in the protein database. Thus, we modeled the structure of this protein using a homology modeling approach [56]. The amino acid sequence of LuxS (*S. pyogenes*) was retrieved from the Uniprot database (Uniprot id: P0C0C7). The structure for this protein was generated, taking the available LuxS from *Streptococcus suis* (pdb id: 4XCH) as a template. This protein shares an identity of 81% with LuxS (*S. pyogenes*). Modeller9v14 was also employed to generate a structure of LuxS (*S. pyogenes*) by homology modeling [57]. Discrete optimized protein energy (DOPE) scores were used to select the best of the five models of LuxS generated. The generated structure was further subjected to validation using Procheck [58].

#### 4.11.2. Molecular Docking

The PubChem compound database was used to obtain the 3D structures of Ct and Pt [59]. Molecular docking study was carried out to obtain further insight into Ct and Pt binding within the active site of LuxS, an important protein that regulates pathogenic traits in *S. pyogenes*. Molecular docking of these compounds against LuxS was conducted by Autodock 4.0 using the Lamarckian genetic algorithm [60]. In total, the numbers of runs were set at 15. The binding free energy was taken into considerations to determine the optimal docking.

### 4.12. Statistical Analysis

All the experiments were performed in triplicate. The data for every experiment were summarized as the mean ± standard deviation (SD). ANOVA was used to analyze the data, with a *p*-value of ≤0.05 using the “Proc. Mixed procedure”, of the SAS Institute, Inc. software version 9.11 (SAS Institute, Inc., Cay, NC, USA). For qRT-PCR, one-tailed Student’s *t*-test was used to determine the significance of the difference between the mean expression of a given experimental sample and the control samples. The results with a *p*-value ≤ 0.05 were considered statistically significant.

## Figures and Tables

**Table 1:** The binding free energy and the residues involved in generating hydrogen bonds at each ligand of LuxS protein with citral and phloretin.

Protein	Compound	Binding Free Energy (kcal/mol)	Residues Involved	
			Hydrogen Bond	Hydrogen Bond
LuxS	Citral	−3.82	H57, H61	T53, L56, H57, E60
	Phloretin	−4.97	N129, D132	T53, H57, P79, F80, G81

**Table 2:** The selected genes and the primers used for the qRT-PCR to investigate their expression in relation to *S. pyogenes* biofilm formation and its virulence.

Gene	Description	Primer Sequence (5′-3′)
*gyrA*	Normalizing internal standard; gyrase	F-CTGCCGCTCAACGTTATACT R-ACTGGTTCTCTTTCGCTTCC
*hasA*	hyaluronic acid synthesis	F-AGCGTGCTGCTCAATCATTA R-CATCCCCAATGCTAACAGGT
*ropB*	global transcriptional regulator	F-TGATATGGATACGGCAAAAC R-TTGACCAAGGCAAAAAGGTT
*luxS*	Involved in quorum sensing	F-CCTAGTGCAGCCTAACCAAA R-GGAGAGCAATCAATCATCCC
*speB*	Extracellular protease	F-CTAGGATACTCTACCAGCG R-CAGTAGCAACACATCCTG
*ciaH*	Role in stress responses	F-CATGTTGCGAACCTCGTCTA R-GGCGGTCTTACAGAATCGTC
*srtB*	Involved in aggregation	F-GCTGGTTTTGGTTTGTGGGA R-CCCCGGGATATTTAACCAACC
*36*	D-alanylation of lipotheicoic acid	F-CAATCGGCAAGCGGGTATAA R-ATGCCTATGGACCAACAGAAG
*covR*	Repressor and sensor kinase genes of the *covRS* TCS pathway	F-CGTCTTTCTGAGGTGGACTCTA R-CTAATGACTCGACTGCCCTTTC
*covS*		F-GTCAATGGTCGTGAAGGGTTAG R-CAAACGACGGGTCACTTCAA

## References

[1] Shulman S.T., Bisno A.L., Clegg H.W., Gerber M.A., Kaplan E.L., Lee G., Martin J.M., Beneden C.V. (2012). Clinical practice guideline for the diagnosis and management of group A streptococcal pharyngitis: 2012 update by the Infectious Diseases Society of America. Clin. Infect. Dis..

[2] Ralph A.P., Carapetis J.R. (2013). Group A streptococcal diseases and their global burden. Curr. Top. Microbiol. Immunol..

[3] Tan L.K., Eccersley L.R., Sriskandan S. (2014). Current views of hemolytic streptococcal pathogenesis. Curr. Opin. Infect. Dis..

[4] Henningham A., Barnett T.C., Maamary P.G., Walker M.J. (2012). Pathogenesis of group A streptococcal infection. Discov. Med..

[5] Fiedler T., Köller T., Kreikemeyer B. (2015). *Streptococcus pyogenes* biofilms-formation, biology, and clinical relevance. Front. Cell Infect. Microbiol..

[6] Macé S., Hansen L.T., Rupasinghe H.P.V. (2017). Anti-bacterial activity of phenolic compounds against *Streptococcus pyogenes*. Medicines.

[7] Chahine E.B., Sucher A.J. (2013). Update on the management of streptococcal pharyngitis. US Pharm.

[8] Baldassarri L., Creti R., Recchia S., Imperi M., Facinelli B., Giovanetti E., Pataracchia M., Alfarone G., Orefici G. (2006). Therapeutic failures of antibiotics used to treat macrolide-susceptible *Streptococcus pyogenes* infections may be due to biofilm formation. J. Clin. Microbiol..

[9] Adil M., Rupasinghe H.P.V. (2018). Application of medicinal plants as a source for therapeutic agents against *Streptococcus pyogenes* infections. Curr. Drug Metab..

[10] Spengler G., Kincses A., Gajdács M., Amaral L. (2017). New roads leading to old destinations: Efflux pumps as targets to reverse multidrug resistance in bacteria. Molecules.

[11] Gajdács M. (2019). The concept of an ideal antibiotic: Implications for drug design. Molecules.

[12] Abachi S., Lee S., Rupasinghe H.P.V. (2016). Molecular mechanisms of inhibition of Streptococcus species by phytochemicals. Molecules.

[13] Ortiz M.I., González-García M.P., Ponce-Monter H.A., Castañeda-Hernández G., Aguilar-Robles P. (2010). Synergistic effect of the interaction between naproxen and citral on inflammation in rats. Phytomedicine.

[14] Shi C., Song K., Zhang X., Sun Y., Sui Y., Chen Y., Jia Z., Sun H., Sun Z., Xia X. (2016). Antimicrobial activity and possible mechanism of action of citral against *Cronobacter sakazakii*. PLoS ONE.

[15] Leite M.C., Bezerra A.P., de Sousa J.P., Guerra F.Q., Lima Ede O. (2014). Evaluation of antifungal activity and mechanism of action of citral against *Candida albicans*. Evid Based Complement. Alternat. Med..

[16] Tao O., Yang Q., Jia L. (2014). Citral inhibits mycelial growth of Penicilliumitalicum by a membrane damage mechanism. Food Control..

[17] Gupta P., Patel D.K., Gupta V.K., Pal A., Tandon S., Darokar M.P. (2017). Citral, a monoterpenoid aldehyde interacts synergistically with norfloxacin against methicillin resistant Staphylococcus aureus. Phytomedicine.

[18] Diniz-Silva H.T., Magnani M., de Siqueira S., de Souza E.L., de Siqueira-Júnior J.P. (2017). Fruit flavonoids as modulators of norfloxacin resistance in Staphylococcus aureus that overexpresses norA. LWT-Food Sci. Technol..

[19] Wen Z.T., Burne R.A. (2004). LuxS-mediated signaling in *Streptococcus mutans* is involved in regulation of acid and oxidative stress tolerance and biofilm formation. J. Bacteriol..

[20] Fuqua W.C., Winans S.C., Greenberg E.P. (1994). Quorum sensing in bacteria: The LuxR-LuxI family of cell density-responsive transcriptional regulators. J. Bacteriol..

[21] Kleerebezem M., Quadri L.E., Kuipers O.P., De Vos W.M. (1997). Quorum sensing by peptide pheromones and two-component signal-transduction systems in Gram-positive bacteria. Mol. Microbiol..

[22] Shapiro J.A. (1998). Thinking about bacterial populations as multicellular organisms. Annu. Rev. Microbiol..

[23] Graham M.R., Smoot L.M., Lux Migliaccio C.A., Virtaneva K., Sturdevant D.E., Porcella S.F., Federle M.J., Scott G.J., Musser J.M. (2002). Virulence control in group A streptococci by a two-component gene regulatory system: Global expression profiling and in vivo infection modeling. Proc. Natl. Acad. Sci. USA.

[24] Kang S.O., Caparon M.G., Cho K.H. (2010). Virulence gene regulation by CvfA, a putative RNase: The CvfA-enolase complex in *Streptococcus pyogenes* links nutritional stress, growth-phase control, and virulence gene expression. Infect. Immun..

[25] Lyon W.R., Madden J.C., Stein J., Caparon M.G. (2001). Mutation of luxS affects growth and virulence factors expression in *Streptococcus pyogenes*. Mol. Microbiol..

[26] Beema S.R.M., Selvaraj C., Singh S.K., Karutha P.S. (2014). *In silico* and in vitro studies of cinnamaldehyde and their derivatives against LuxS in *Streptococcus pyogenes*: Effects on biofilm and virulence genes. J. Mol. Recognit..

[27] LaSarre B., Federle M.J. (2013). Exploiting quorum sensing to confuse bacterial pathogens. Microbiol. Mol. Biol. Rev..

[28] Magi G., Marini E., Facinelli B. (2015). Antimicrobial activity of essential oils and carvacrol, and synergy of carvacrol and erythromycin, against clinical, erythromycin-resistant Group A Streptococci. Front. Microbiol..

[29] Wijesundara N.M., Sekhon-Loodu S., Rupasinghe H.P.V. (2017). Phytochemical-rich medicinal plant extracts suppress bacterial antigens-induced inflammation in human tonsil epithelial cells. Peer J..

[30] Hasan S., Danishuddin M., Adil M., Singh K., Verma P.K., Khan A.U. (2012). Efficacy of *E. officinalis* on the cariogenic properties of *Streptococcus mutans*: A novel and alternative approach to suppress quorum-sensing mechanism. PLoS ONE.

[31] Schifferli D.M., Abraham S.N., Beachey E.H. (1986). Influence of trimethoprim and sulfamethoxazole on the synthesis, expression, and function of type 1 fimbriae of Escherichia coli. J. Infect. Dis..

[32] Sun D., Courtney H.S., Beachey E.H. (1988). Berberine sulfate blocks adherence of *Streptococcus pyogenes* to epithelial cells, fibronectin, and hexadecane. Antimicrob. Agents Chemother..

[33] Stiefel P., Schmidt-Emrich S., Maniura-Weber K., Ren Q. (2015). Critical aspects of using bacterial cell viability assays with the fluorophores SYTO9 and propidium iodide. BMC Microbiol..

[34] Adil M., Singh K., Verma P.K., Khan A.U. (2014). Eugenol-induced suppression of biofilm-forming genes in *Streptococcus mutans*: An approach to inhibit biofilms. J. Glob. Antimicrob. Resist..

[35] Chaussee M.A., Callegari E.A., Chaussee M.S. (2004). Rgg regulates growth phase-dependent expression of proteins associated with secondary metabolism and stress in *Streptococcus pyogenes*. J. Bacteriol..

[36] Falaleeva M., Zurek O.W., Watkins R.L., Reed R.W., Ali H., Sumby P., Voyich J.M., Korotkova N. (2014). Transcription of the *Streptococcus pyogenes* hyaluronic acid capsule biosynthesis operon is regulated by previously unknown upstream elements. Infect. Immun..

[37] Viszwapriya D., Subramenium G.A., Prithika U., Balamurugan K., Pandian S.K. (2016). Betulin inhibits virulence and biofilm of *Streptococcus pyogenes* by suppressing ropB core regulon, sagA and dltA. FEMS Path. Dis..

[38] Shi C., Zhao X., Liu Z., Meng R., Chen X., Guo N. (2016). Antimicrobial, antioxidant, and antitumor activity of epsilon-poly-l-lysine and citral, alone or in combination. Food Nutr. Res..

[39] Nithiya T., Udayakumar R. (2016). In vitro antioxidant properties of phloretin-an important phytocompound. J. Biosci. Med..

[40] Tatsuno I., Isaka M., Okada R., Zhang Y., Hasegawa T. (2014). Relevance of the two-component sensor protein CiaH to acid and oxidative stress responses in *Streptococcus pyogenes*. BMC Res. Notes.

[41] Kapur V., Topouzis S., Majesky M.W., Li L.L., Hamrick M.R., Hamill R.J., Patti J.M., Musser J.M. (1993). A conserved *Streptococcus pyogenes* extracellular cysteine protease cleaves human fibronectin and degrades vitronectin. Microb. Pathog..

[42] Terao Y. (2012). The virulence factors and pathogenic mechanisms of *Streptococcus pyogenes*. J. Oral. Biosci..

[43] Miörner H., Johansson G., Kronvall G. (1983). Lipoteichoic acid is the major cell wall component responsible for surface hydrophobicity of group A streptococci. Infect. Immun..

[44] Cox K.H., Ruiz-Bustos E., Courtney H.S., Dale J.B., Pence M.A., Nizet V., Aziz R.K., Gerling I., Price S.M., Hasty D.L. (2009). Inactivation of DltA modulates virulence factor expression in *Streptococcus pyogenes*. PLoS ONE.

[45] Nandu T.G., Subramenium G.A., Shiburaj S., Viszwapriya D., Iyer P.M., Balamurugan K., Pandian S.K. (2018). Fukugiside, a biflavonoid from *Garcinia travancorica* inhibits biofilm formation of *Streptococcus pyogenes* and its associated virulence factors. J. Med. Microbiol..

[46] Marouni M.J., Sela S. (2003). The luxS gene of *Streptococcus pyogenes* regulates expression of genes that affect internalization by epithelial cells. Infect. Immun..

[47] Brackman G., Quntar A.A., Enk C.D., Karalic I., Nelis H.J., Van Calenbergh S., Srebnik M., Coenye T. (2013). Synthesis and evaluation of thiazolidinedione and dioxazaborocane analogues as inhibitors of AI-2 quorum sensing in *Vibrio harveyi*. Bioorg. Med. Chem..

[48] Li M., Ni N., Chou H.T., Lu C.D., Tai P.C., Wang B. (2008). Structure-based discovery and experimental verification of novel AI-2 quorum sensing inhibitors against *Vibrio harveyi*. Chem. Med. Chem..

[49] Zhu P., Li M. (2012). Recent progresses on AI-2 bacterial quorum sensing inhibitors. Curr. Med. Chem..

[50] Wu G.Q., Yang Q., Yang Q., Long M., Guo L., Li B., Meng Y., Zhang A., Wang H., Liu S. (2015). Evaluation of agar dilution and broth microdilution methods to determine the disinfectant susceptibility. J. Antibiot..

[51] Zutkis A.A., Anbalagan S., Chaussee M.S., Dmitriev A.V. (2014). Inactivation of the Rgg2 transcriptional regulator ablates the virulence of *Streptococcus pyogenes*. PLoS ONE.

[52] Courtney H.S., Ofek I., Penfound T., Nizet V., Pence M.A., Kreikemeyer B., Podbielski A., Hasty D.L., Dale J.B. (2009). Relationship between expression of the family of M proteins and lipoteichoic acid to hydrophobicity and biofilm formation in *Streptococcus pyogenes*. PLoS ONE.

[53] Bortolin M., De Vecchi E., Romano` C.A., Toscano M., Mattina R., Drago L. (2016). Antibiofilm agents against MDR bacterial strains: Is bioactive glass BAG-S53P4 also effective?. J. Antimicrob. Chemother..

[54] Khan R., Adil M., Danishuddin M., Verma P.K., Khan A.U. (2012). In vitro and in vivo inhibition of *Streptococcus mutans* biofilm by *Trachyspermum ammi*: An approach of alternative medicine. Phytomedicine.

[55] Schmittgen T.D., Liyak K.J. (2008). Analyzing real-time PCR data by the comparative CT method. Nat. Protoc..

[56] Rodriguez R., Chinea G., Lopez N., Pons T., Vriend G. (1998). Homology modeling, model and software evaluation: Three related resources. Bioinformatics.

[57] Webb B., Sali A. (2017). Protein structure modeling with MODELLER. Methods Mol. Biol..

[58] Laskowski R.A., MacArthur M.W., Moss D.S., Thornton J.M. (1993). PROCHECK: A program to check the stereochemical quality of protein structures. J. Appl. Cryst..

[59] Kim S., Thiessen P.A., Bolton E.E., Chen J., Fu G., Gindulyte A., Wang J., Yu B., Zhang J., Bryant S.H. (2016). PubChem substance and compound databases. Nucleic Acids Res..

[60] Goodsell D.S., Morris G.M., Olson A.J. (1996). Automated docking of flexible ligands: Applications of AutoDock. J. Mol. Recognit..

